# CK2 Inhibition Prior to Status Epilepticus Persistently Enhances K_Ca_2 Function in CA1 Which Slows Down Disease Progression

**DOI:** 10.3389/fncel.2020.00033

**Published:** 2020-02-26

**Authors:** Felix Schulze, Steffen Müller, Xiati Guli, Lukas Schumann, Hannes Brehme, Till Riffert, Marco Rohde, Doreen Goerss, Simone Rackow, Anne Einsle, Timo Kirschstein, Rüdiger Köhling

**Affiliations:** ^1^Oscar Langendorff Institute of Physiology, University of Rostock, Rostock, Germany; ^2^Department of Psychosomatic Medicine, Rostock University Medical Center, Rostock, Germany; ^3^German Center for Neurodegenerative Diseases (DZNE), Göttingen, Germany; ^4^Center of Transdisciplinary Neurosciences Rostock, University of Rostock, Rostock, Germany

**Keywords:** temporal lobe epilepsy, patch-clamp, intracellular recording, field potential recording, video-EEG monitoring, Timm stain

## Abstract

**Purpose:**

Epilepsy therapy is currently based on anti-seizure drugs that do not modify the course of the disease, i.e., they are not anti-epileptogenic in nature. Previously, we observed that *in vivo* casein kinase 2 (CK2) inhibition with 4,5,6,7-tetrabromotriazole (TBB) had anti-epileptogenic effects in the acute epilepsy slice model.

**Methods:**

Here, we pretreated rats with TBB *in vivo* prior to the establishment of a pilocarpine-induced status epilepticus (SE) in order to analyze the long-term sequelae of such a preventive TBB administration.

**Results:**

We found that TBB pretreatment delayed onset of seizures after pilocarpine and slowed down disease progression during epileptogenesis. This was accompanied with a reduced proportion of burst firing neurons in the CA1 area. Western blot analyses demonstrated that CA1 tissue from TBB-pretreated epileptic animals contained significantly less CK2 than TBB-pretreated controls. On the transcriptional level, TBB pretreatment led to differential gene expression changes of K_Ca_2.2, but also of HCN1 and HCN3 channels. Thus, in the presence of the HCN channel blocker ZD7288, pretreatment with TBB rescued the afterhyperpolarizing potential (AHP) as well as spike frequency adaptation in epileptic animals, both of which are prominent functions of K_Ca_2 channels.

**Conclusion:**

These data indicate that TBB pretreatment prior to SE slows down disease progression during epileptogenesis involving increased K_Ca_2 function, probably due to a persistently decreased CK2 protein expression.

## Introduction

Temporal lobe epilepsy (TLE) is the most common focal symptomatic epileptic disorder in the adulthood and typically difficult to treat (Kwan and Brodie, 2000; Blümcke et al., 2013). Moreover, all currently available drugs used to treat focal symptomatic epilepsy are merely anti-seizure drugs, i.e., they are anti-ictogenic in nature, but lack any anti-epileptogenic potential (Schmidt and Sillanpää, 2016). In the last decades, several rodent post-status epilepticus (SE) models such as the pilocarpine animal model have provided valuable insights into the latent period between the initial insult and the subsequent development of chronic TLE (Leite et al., 2002). During this period of epileptogenesis, marked changes in synaptic and intrinsic properties take place that may facilitate the occurrence of seizures—most profoundly and studied at best—within the hippocampal network (Leite et al., 2002).

Among these changes, it appears that channels that are active near the resting membrane potential (RMP) such as K^+^ or hyperpolarization-activated cyclic nucleotide-gated non-selective (HCN) channels are particularly intriguing candidates involved in epileptogenesis. With respect to the CA1 area in the pilocarpine model, there is evidence of persistent downregulation of K_*v*_4.2 (Bernard et al., 2004), K_Ca_2.2 (Schulz et al., 2012), and HCN1 (Jung et al., 2007, 2010). Importantly, these transcriptional changes partly seem to involve the activation of protein kinases or phosphatases (Bernard et al., 2004; Jung et al., 2010). In turn, protein kinase and/or phosphatase activation also seem to be involved in acute epileptic conditions (Jung et al., 2010; Kernig et al., 2012) suggesting that neuronal hyperactivity may trigger these enzymatic pathways.

Casein kinase 2 (CK2) is a widely expressed enzyme with a substantial number of possible targets; however, one of its major targets is calmodulin (CaM) and K_Ca_2.2 (Bildl et al., 2004; Allen et al., 2007). We recently demonstrated that oral administration of the CK2 blocker 4,5,6,7-tetrabromotriazole (TBB) enhanced K^+^ currents mediating the afterhyperpolarizing potential (AHP) in the pilocarpine model, and even more intriguing, blocked the occurrence of spontaneous epileptic activity in the acute slice model with Mg^2+^ removal (Brehme et al., 2014). This compound obviously exerted an anti-epileptogenic potential in the *in vitro* slice preparation, but is not an anti-seizure drug in this model *in vivo* (Bajorat et al., 2018). Therefore, we asked whether TBB might have disease modifying effects in the pilocarpine model *in vivo*, too. To this end, we treated the rats with TBB prior to pilocarpine-induced SE and analyzed the animals in their chronically epileptic stage.

## Materials and Methods

### TBB Pretreatment and Pilocarpine-Induced Chronic Epilepsy

**FIGURE 1:**
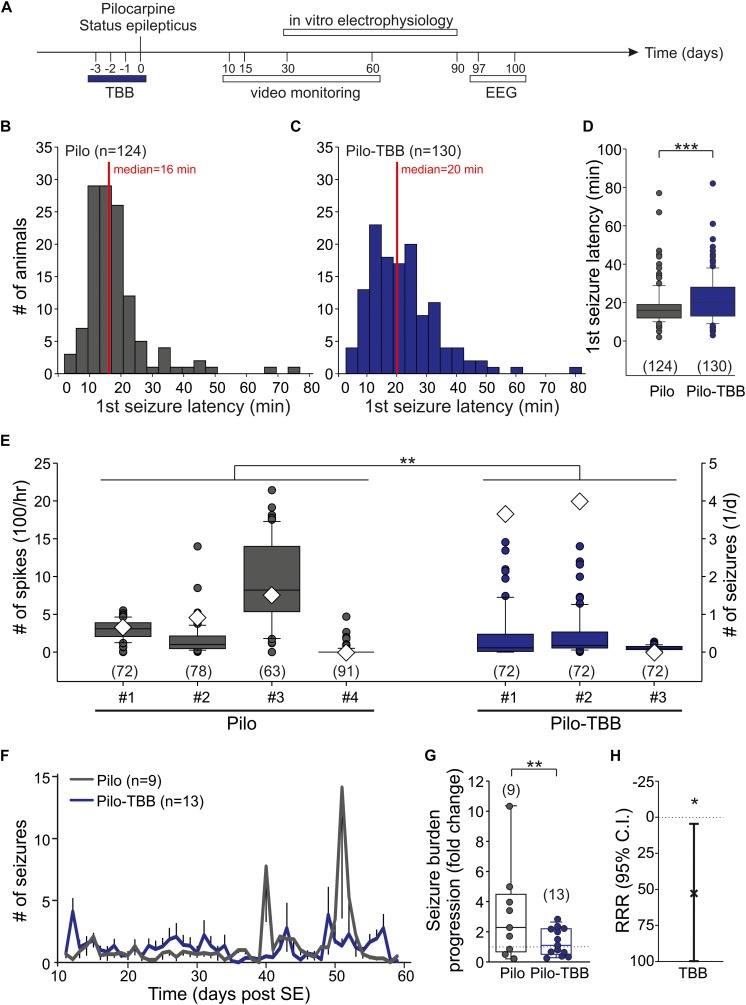
CK2 inhibition exerts disease modifying properties. **(A)** Time frame of the experiment. TBB was administered four times *in vivo* on days “−3” (i.e., 3 days before status epilepticus) through “0” (i.e., day of pilocarpine-induced status epilepticus). All experiments thereafter were performed during the chronic stage of recurrent seizures. **(B–D)** Latency to the first stage 4 or 5 seizure after pilocarpine injection in vehicle-pretreated **(B)** and TBB-pretreated **(C)** rats. The median latency was significantly (***P < 0.001, Mann-Whitney U-test) prolonged in the TBB-pretreated group **(D)**. **(E)** Number of interictal spikes per hour (left *y*-axis) in the electroencephalogram (EEG) during 3 days (97–100 post-SE). Note that the number of daily seizures during this period is also depicted (diamonds, right *y*-axis). There was a significant group effect of TBB pretreatment (**P < 0.01, two-way ANOVA with Tukey *post hoc* test). **(F)** Number of generalized seizures (at least stage 3) at daylight (from 06:00 to 18:00 h) for both animal groups as grand average for the entire recording period. **(G)** Seizure burden progression as fold change of seizure rates during epileptogenesis. The variability was significantly reduced in TBB-pretreated epileptic animals (**P < 0.01, *F*-test). **(H)** Relative risk reduction (RRR) of seizure progression by TBB pretreatment was significant (*P < 0.05, *t*-test).

Male Wistar rats (27–30 days; Charles River, Sulzfeld, Germany) were treated with the CK2 inhibitor TBB (Tocris Bioscience, Bristol, United Kingdom or Sigma–Aldrich, Taufkirchen, Germany) 3 days before SE by intraperitoneal (i.p.) injection of 6 μmol/kg body weight [stock solution 6 mmol/l, dissolved in dimethylsulfoxide (DMSO), 1 μl/g body weight]. This was repeated for three times every 24 h (i.e., four consecutive daily injections, Figure 1A). The same amount of vehicle (DMSO, 1 μl/g body weight) was injected as control. Allocation of animals to the TBB or vehicle pretreatment group was performed in randomized fashion. Following the last of four injections, the animals were allowed to recover for one hour, before pilocarpine was injected to induce chronic epilepsy. During the TBB pretreatment period, the weight gain was monitored and did not differ significantly between TBB-treated (from 105 ± 18 to 131 ± 19 g, mean ± SD) and vehicle-treated (from 107 ± 19 to 133 ± 18 g, mean ± SD) animals (ANOVA).

Chronic epilepsy was induced by systemic pilocarpine injection 1 h after the last TBB application. As described previously (Kirschstein et al., 2007; Bajorat et al., 2011; Müller et al., 2013), rats received *N*-methyl scopolamine nitrate (1 mg/kg, i.p.) in order to reduce peripheral cholinergic effects, and then pilocarpine hydrochloride (340 mg/kg, i.p.). All pilocarpine-injected animals were carefully monitored to detect spontaneous motor seizures with progression into limbic SE. All stage 4 or 5 seizures (Racine, 1972) were registered for each individual animal to detect the latency to the first stage 4 or 5 seizure after pilocarpine. The onset of SE was determined when an animal had a stage 4 or 5 seizure (Racine, 1972) that was followed by continuous seizure motor activity without showing any reaction to sensory stimuli such as gently touching against the whiskers. SE was terminated after 40 min by administration of diazepam solution (Ratiopharm, Ulm, Germany, 5 mg/ml, i.p.). Finally, the rats were fed with 5% glucose solution for 1 day and kept in separate cages. Experimenters were blinded for all *in vivo* applications and observations, thus were unaware as to whether the animal was pretreated with TBB or vehicle (DMSO) before.

All procedures were performed according to national and international guidelines on the ethical use of animals (European Council Directive 86/609/EEC, approval of local authority LALLF M-V/TSD/7221.3-1.1-013/10 and 7221.3-1.1-019/13), and all efforts were made to minimize animal suffering and to reduce the number of animals used.

### Video-EEG Recording and Video Monitoring

Epileptiform potentials in the electroencephalogram (EEG) were recorded using a telemetric system [Neuroscore 2.0, Data Science International (DSI), Arden Hills, MN, United States]. Electrode implantation (TA10EA-F20 or TA11CA-F40 from DSI) was performed as previously described (Bajorat et al., 2011, 2016, 2018). Video-EEG monitoring was performed in three TBB-treated pilocarpine-treated epileptic animals and four vehicle-treated epileptic rats under environmentally controlled conditions (12 h light/dark cycles, lights switched on from 6 a.m. to 6 p.m., 22 ± 2°C, and 40–60% relative humidity in an isolated room). Pilocarpine-treated rats were housed in individual cages and continuous video-EEG monitoring was performed using a telemetric system (Dataquest A.R.T. 4.2., DSI) in combination with a light and dark network camera (Axis 223M, Axis communications, Lund, Sweden). Night recordings were performed with a small blue night light for residual light intensification. The EEG was screened for epileptiform potentials (spikes, sharp waves, spike-and-wave-complexes, sharp-and-slow-wave-complexes) and seizures were detected by concomitant analysis of the video signal (Bajorat et al., 2011, 2016). The EEG experimenter was blinded to the treatment (TBB versus vehicle).

In addition, we performed video analysis in order to detect generalized seizures (13 TBB-treated and nine vehicle-treated epileptic rats) for 7 weeks (10–60 days after pilocarpine-induced SE, i.e., 5–12 weeks of age). All generalized seizures (stage 4–5, Racine, 1972) at daylight (from 6 a.m. to 6 p.m.) were detected in a blinded fashion, i.e., without knowledge about the treatment (TBB versus vehicle). Control animals, i.e., animals that received saline instead of pilocarpine, were repeatedly reported to get no spontaneous seizures at all (Bajorat et al., 2011, 2016) and were therefore not included in these *in vivo* studies. The seizure burden progression was calculated as the ratio of seizure rate day 35–59 over the seizure rate day 11–20. The relative risk reduction (RRR) of seizure burden progression was calculated as 1 – the relative risk (seizure burden progression in the TBB-pretreated animal over the seizure burden progression in the vehicle-pretreated group).

### Tissue Preparation

Hippocampal slices were prepared using 8–12-week-old animals (i.e., 4–8 weeks after SE). At this age, chronically epileptic animals show on average 4.5 seizures per day (Bajorat et al., 2011). After deep anesthesia with diethyl ether (Merck, Darmstadt, Germany), rats were decapitated and the brain was rapidly removed and submerged into oxygenated ice-cold dissection solution containing (in mM) 125 NaCl, 26 NaHCO_3_, 3 KCl, 1.25 NaH_2_PO_4_, 0.2 CaCl_2_, 5 MgCl_2_, and 13 D-glucose (95% O_2_, 5% CO_2_; pH 7.4; osmolality 306–314 mosmol/kg H_2_O). Hippocampal horizontal brain slices (400 μm) were prepared from the brain using a vibratome (Leica VT1200S, Wetzlar, Germany), and these slices were gently transferred into a holding chamber containing artificial cerebrospinal fluid (ACSF) of the following composition: (in mM) 125 NaCl, 26 NaHCO_3_, 3 KCl, 1.25 NaH_2_PO_4_, 2.5 CaCl_2_, 1.3 MgCl_2_, and 13 D-glucose (95% O_2_, 5% CO_2_; pH 7.4; osmolality 306–314 mosmol/kg H_2_O).

For electrophysiological experiments, hippocampal slices were allowed to recover at room temperature (20–22°C) for at least 1 h before being transferred into recording chamber. For quantitative real-time reverse transcriptase PCR (RT-PCR) analyses, hippocampal slices were immediately dissected into mini-slices. For Timm staining, hippocampal slices were immediately immersed into Na_2_S solution.

### Electrophysiological Experiments

#### Extracellular Recordings

After recovery, hippocampal slices were placed into an interface chamber continuously bathed with ACSF at a flow rate of 2 ml/min using a volumetric infusion pump (MCM-500, MC Medicine technique GmbH, Alzenau, Germany). Bath temperature was maintained at 32 ± 1°C by (npi electronic GmbH, Tamm, Germany), and all experiments started after an equilibration time of at least 30 min. Schaffer collateral fibers were stimulated using bipolar platinum wire electrodes connected to an A365 stimulus isolator in constant-current mode (Word Precision Instruments, Friedberg, Germany) and driven by the Master-8 stimulator (A.M.P.I., Jerusalem, Israel). The stimulating electrode was placed into stratum radiatum at the border between CA2 and CA1. Field excitatory postsynaptic potentials (fEPSPs) from the CA1 area were recorded using borosilicate glass pipettes (2–3 MΩ, pulled with PIP5 from HEKA Elektronik, Lambrecht, Germany; filled with ACSF). Data were acquired using a Micro1401 A/D converter and signal 2.16 software (CED, Cambridge Electronic Design, Cambridge, United Kingdom). The fEPSP slope was determined as the local minimum of the differentiated fEPSP curve indicating the maximal negative gradient during its falling phase. With these extracellular recordings, we obtained the input–output curve, the paired-pulse ratio (PPR) with an interstimulus interval of 40 ms and short-term plasticity data.

#### Patch-Clamp Experiments

Patch pipettes with a tip resistance 3–6 MΩ were pulled from borosilicate glass capillaries (GB150-8P; Science Products GmbH, Hofheim, Germany) using a horizontal electrode puller (P-97 Micropipette Puller; Sutter Instrument Company, Novato, CA, United States) and filled with an intracellular solution (K^+^ gluconate 140, MgCl_2_ 2, HEPES 10, phosphocreatine 10, Na_2_-ATP 2, Na_2_-GTP 0.4, pH 7.3 adjusted with KOH, osmolality 290–300 mosmol/kg H_2_O). Whole-cell patch-clamp recordings from visualized CA1 pyramidal cells were performed in the slice as previously described (Müller et al., 2013) at room temperature using an upright microscope (Nikon Eclipse FN1, Düsseldorf, Germany) and a standard amplifier (ELC-03XS, npi electronic, Tamm Germany, run with signal 2.16 software, CED).

Signals were low-pass filtered at 2 kHz and digitized at a sampling frequency of 4 kHz. Only cells with an RMP of −50 mV or more negative were included in this study. The AHP-mediating K^+^ current and charge transfer were measured in the voltage-clamp mode as previously described (Brehme et al., 2014). In brief, the recording solution contained 500 nM tetrodotoxin (TTX) and 1 mM tetraethylammonium (TEA) (both from Tocris Bioscience, Bristol, United Kingdom) to block voltage-activated Na^+^ and K^+^ channels, respectively. AHP-mediating currents were elicited by a depolarizing voltage command from the holding potential −60 to +60 mV for 100 ms, followed by a step to −120 mV for 2 ms and a return to −60 mV for 2800 ms. The medium AHP (mAHP) was determined as the peak outward current during the first 400 ms following the short hyperpolarizing step to −120 mV (2 ms) and normalized to cell capacitance. The slow component of the AHP (sAHP) was assessed by determining the charge transfer, i.e., the area under the curve (AUC), for the interval between 400 and 2400 ms (i.e., 2000 ms), also normalized to cell capacitance. Low and fast capacitive current transients as well as leak currents were fully compensated and monitored (Cf and Cs). Series resistance ranged from 4 to 15 MΩ and was monitored at regular intervals throughout the experiments.

#### Intracellular Recordings

Intracellular recordings were performed in CA1 pyramidal cells impaled with borosilicate glass microelectrodes (80–120 MΩ, pulled with Sutter P-97 and filled with 3 M potassium acetate and 0.013 M KCl) using an SEC-10LX amplifier (npi electronic). In these recordings, RMP, membrane resistance, and membrane time constant were determined. The membrane resistance was calculated as the slope of the steady-state current–voltage curve obtained by hyperpolarizing current injection (ranging from −1.0 to −0.2 nA for 400 ms). The membrane time constant was calculated as the average time constant during the hyperpolarizing steps. Subsequent depolarizing current injection (from + 0.1 to + 0.5 nA) was used to evoke a train of action potentials (400 ms). The AHP following this prolonged depolarization (referred to as train-AHP) was calculated as the difference between the train-AHP peak amplitude and the RMP (for which the last sweep with + 0.5 nA depolarization was used). In addition, burst firing was tested with short depolarizing current steps (duration 3–7 ms, from + 0.5 to + 2.4 nA). Unless otherwise indicated, chemicals were purchased from Sigma–Aldrich (Taufkirchen, Germany).

### Quantitative Real-Time Reverse Transcriptase PCR Analyses

Hippocampal horizontal slices were immediately transferred into ice-cold phosphate-buffered saline (PBS, pH = 7.4, PAA Laboratories GmbH, Austria), and the CA1 region was dissected out (referred to as mini-slice) under a binocular microscope (Leica). All mini-slices from one animal (*n* = 6–8) were pooled and immediately frozen in liquid nitrogen. For mRNA isolation, TRIZOL reagent was used, and total RNA was reverse-transcribed using Moloney murine leukemia virus reverse transcriptase (final concentration [Cf] = 10 U/μl) and RNasin Plus RNase inhibitor (Cf = 2 U/μl, both Promega Corporation, Madison, WI, United States) in the presence of random hexamers (Cf = 0.01 μg/μl) and dNTP Mix (Cf = 0.5 nmol/μl each, Invitrogen, Carlsbad, CA, United States). For the real-time PCR of the target genes (K_Ca_2.2, HCN1, HCN2, HCN3, and HCN4) as well as three standard reference genes (phosphoglycerate kinase 1, S18 ribosomal protein, 18S rRNA), we used the QuantiFast SYBR Green PCR Kit (concentration as recommended by the manufacturer, Qiagen Inc., Valencia, CA, United States). The mastermix was aliquoted, cDNA (Cf = 0.5 ng/μl) and primers (Cf = 0.5 pmol/μl; TIB Molbiol, Berlin, Germany) were added.

Real-time PCRs were performed using the ep mastercycler (software realplex 2.2, Eppendorf, Hamburg, Germany) with cycling parameters as follows: 95°C for 5 min once, followed by 95°C for 15 s and 60°C for 15 s, with normalized fluorescence read at 68°C (530 nm) for 40 cycles. To confirm single product amplification, melting curve analysis and gel electrophoresis were done. Expression levels of ion channel mRNA were normalized to all three housekeeping genes (TIB Molbiol, Berlin, Germany) and analyzed by 2^–^^ΔΔ^^*Ct*^ method (i.e., normalized to control levels).

### Western Blot

For CK2 western blot analysis, six control and six epileptic TBB-pretreated animals were used at age 133/134 days. Pools of mini-slices, each for CA1, CA3, and DG area from individual animals were subjected to protein extraction with RIPA buffer [50 mM Tris, 150 mM NaCl, 1% (v/v) TritonX-100, 1% (w/v) sodium deoxycholate, 0.1% (w/v) SDS, 1 mM EDTA] in the presence of Complete protease inhibitor cocktail (Roche). After centrifugation at 14,000 *g* at 4°C for 20 min, 10 μg of total proteins from each sample were separated by 10% SDS-PAGE and blotted onto a PVDF membrane (Immobilon-FL; Millipore). Primary antibody incubation (mouse monoclonal anti-CK2, 1:2000) was done overnight at 4°C and secondary antibody incubation (goat anti-mouse IRDye 680RD, Odyssey, 1:5000) was done for 30 min at room temperature. A single specific protein band was visualized with the Odyssey infrared imaging scanner from Li-cor.

For quantification purposes, GAPDH was used as an internal control for CK2 normalization. Briefly: after CK2 detection, membranes were washed 15 min at 55°C with a buffer (pH 2.2) containing 1.5% glycine, 0.1% SDS, 1% Tween 20 to remove any bound anti-CK2 antibody. Then, according to CK2 immunoreaction, the rabbit monoclonal anti-GAPDH primary antibody (Cell Signaling; 1:5000) and a secondary antibody (goat anti-rabbit IRDye 800CW, Odyssey; 1:5000) was loaded. For each sample, CK2 and GAPDH fluorescence signals were determined with Image Studio lite software (Li-cor) and the ratio between CK2 and GAPDH was calculated.

### Mossy Fiber Sprouting Analysis

Timm staining (Timm, 1958) which typically demonstrates the zinc-containing mossy fibers was done for confirmation of mossy fiber sprouting (MFS) using a previously published protocol (Müller et al., 2014). After incubation the brain section in disodium sulfide solution (9.7 mM Na_2_S, 19.8 mM NaH_2_PO_4_, in 500 ml distilled H_2_O) the sections were fixed in glutaraldehyde solution (100 ml PBS 0.15 M; 1.2 ml 25% glutaraldehyde) for 10 min and subsequently placed in a 30% sucrose solution at 4°C until the brain pieces sank to the bottom of the chamber. Transversal cryosections were cut at 20 μm using a freezing microtome (Leica CM 3050S, Leica Microsystems, Wetzlar, Germany). After washing for 15 min with PBS, the sections were processed in the dark for 30 min in developer solution (80 ml distilled H_2_O; 120 ml gum arabicum; 132.7 mM citric acid; 120.7 mM sodium citrate; 154.4 mM hydroquinone; only in the dark: 0.16 mM AgNO_3_ in distilled H_2_O) and washed with distilled H_2_O. After short incubation with the stop solution (63.25 mM Na_2_S_2_O_3_ in distilled H_2_O), the sections were again washed with distilled H_2_O. Finally, the nuclei were counter-stained (8.17 mM toluidine blue and 49.7 mM Na_2_B_4_O_7_ in distilled H2O). Following washing, the sections from the hippocampi were dehydrated in alcohol and mounted on slides with slide mounting medium (Fluoromount-G^TM^, Beckman-Coulter, Fullerton, CA, United States).

Mossy fiber sprouting was assessed as previously reported (Müller et al., 2014). Briefly, we used a semi-quantitative classification scheme performed by one investigator (TR) blinded with respect to other data as well as to the vehicle or TBB pretreatment (0 = no staining, 1 = patches of staining in the inner molecular layer, 2 = confluent staining, 3 = massive staining). Second, a morphometric analysis measuring the brownish-colored area within the inner molecular layer (MFS area) normalized to the granule cell area in the same slice to produce an MFS ratio (mean values of three sections per animal).

### Statistical Analysis

Unless otherwise indicated, data were expressed as mean values ± the standard error of the mean (SEM). For statistical comparisons, however, data were tested for normal distribution, and then analyses were performed using the appropriate test as indicated. The level of significance was set to *P* < 0.05, and significant differences were indicated with asterisks in all figures (^∗^*P* < 0.05, ^∗∗^*P* < 0.01, ^∗∗∗^*P* < 0.001).

## Results

### TBB Exerts Anti-epileptogenic Properties *in vivo*

The aim of this study was to investigate the acute and long-term effect of CK2 inhibition *in vivo* on the pilocarpine-induced SE and the post-SE chronic epilepsy. To this end, we administered TBB three days before pilocarpine-induced SE, i.e., four consecutive injections (Figure 1A). One hour after the last TBB injection, we started the pilocarpine procedure. Roughly 85% of the animals developed stage 4 or 5 seizures after a single pilocarpine injection. The proportions of rats developing seizures were not influenced by TBB pretreatment (Table 1). In contrast, the median latency of the first stage 4 or 5 seizure after pilocarpine was significantly prolonged from 16 min (Figure 1B) in vehicle-pretreated rats to 20 min in TBB-pretreated animals (*P* < 0.001, Mann–Whitney *U*-test; Figures 1C,D). Other parameters such as number of seizures before SE, diazepam dose, SE rate, and mortality did not differ between both groups (Table 1). Hence, TBB pretreatment prolonged the latency to the first generalized seizure, but did not alter SE rate or mortality.

**TABLE 1:** Status epilepticus (SE) *in vivo* data in vehicle-pretreated and TBB-pretreated epileptic animals.

	**Vehicle-pretreated epileptic rats (Pilo)**	**TBB-pretreated epileptic rats (Pilo-TBB)**
# of stage 4 or 5 seizures before SE	2.4 ± 0.1(*n* = 124)	2.5 ± 0.1(*n* = 130)
Latency of SE (min)	27.4 ± 1.5(*n* = 69)	31.0 ± 1.9(*n* = 79)
Diazepam dose (mg)	3.6 ± 0.2(*n* = 69)	3.4 ± 0.1(*n* = 79)
SE rate	69/146(47.3%)	79/157(50.3%)
Died before/during SE	43/146(29.4%)	36/157(22.9%)
Non-responder to Pilo	34/146(23.3%)	42/157(26.8%)

*There were no significant differences between both groups (Mann–Whitney *U*-test and χ^2^ test, respectively).*

Next, we asked whether pretreatment with TBB could influence epileptogenesis and thus we analyzed animals in their chronic stages of TLE, i.e., at a time-point of fully established epileptogenesis. To this end, we implanted a telemetric EEG system and counted the number of spikes/hour within 3 days (97–100 days post-SE; see timescale in Figure 1A) of four vehicle-pretreated and three TBB-pretreated epileptic animals. Overall, we found 314 ± 25 spikes/hour in vehicle-pretreated rats (in 304 animal hours), which was significantly higher than 168 ± 19 spikes/hour in TBB-pretreated rats (in 216 animal hours; *P* < 0.01, two-way ANOVA followed by Tukey test; Figure 1E). During this period, we detected 32 generalized seizures (23 in TBB-pretreated and nine in vehicle-pretreated animals; see diamonds in Figure 1E). Hence, the interictal spike counts were quite representative and not taken from a period with increased seizure rate. Since the clinical course of chronic epilepsy in this model is often associated with an increasing seizure rate and also with seizure clustering (Bajorat et al., 2011, 2016, 2018), this short-term analysis of EEG-detected seizures cannot reflect seizure burden and hence is insufficient to study the TBB effect.

Therefore, we performed a long-term video analysis of daytime seizures from nine vehicle-pretreated and 13 TBB-pretreated littermates for 7 weeks covering the full period of epileptogenesis (10–60 days after SE; Figures 1F–H). Over this period, vehicle-pretreated animals showed 1.3 ± 1.0 seizures/12 h (*n* = 9) during daylight, i.e., between 6 a.m. and 6 p.m., consistent with previous data (Bajorat et al., 2011, 2016, 2018). TBB-pretreated littermates showed the same overall seizure rates (1.3 ± 1.3 seizures/12 h,*n* = 13), but developed gross changes in seizure rates during the second half of the observation period (Figure 1F). Seizure clusters were present in 6/9 vehicle-pretreated animals, but only in 4/13 TBB-pretreated epileptic animals (*P* = 0.096, χ^2^ test). Disease progression assessed as the ratio of seizure rate during the second half of observation period over the baseline seizure rate was substantially higher in the vehicle-pretreated group as compared to TBB-pretreated animals (314 ± 99% versus 133 ± 24%, *P* = 0.062, *t*-test). Moreover, and probably more importantly, seizure rate variability was significantly reduced by TBB pretreatment (*P* < 0.01, *F*-test; Figure 1G), indicating that the seizure burden was stabilized in the cohort of TBB-treated epileptic rats. Overall, we observed a significant RRR of seizure burden by 58% (95% confidence interval 4–100%, *P* < 0.05, *t*-test, Figure 1H) indicating that disease progression following SE was slowed down in TBB-pretreated epileptic animals.

**FIGURE 2:**
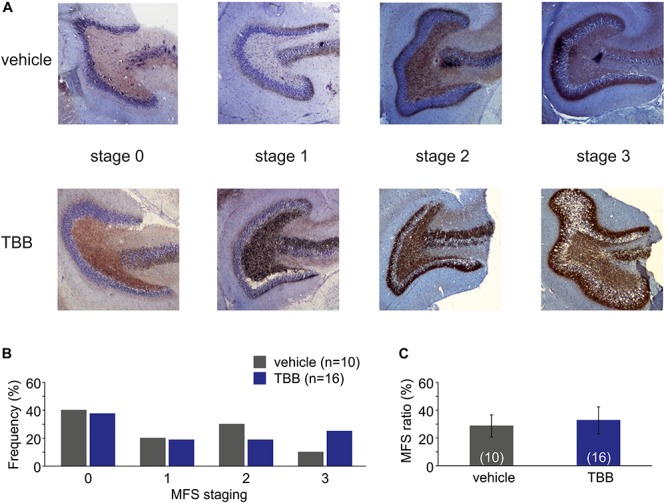
Mossy fiber sprouting (MFS). **(A)** Photomicrographs from vehicle-pretreated epileptic animals (upper row) and TBB-pretreated epileptic animals (lower row) demonstrate the MFS stages 0–3. **(B)** Relative frequencies of MFS stages 0–3 in vehicle- and TBB-pretreated animals. **(C)** MFS ratio (MFS area divided by granule cell area) in both experimental groups.

Next, we asked whether this disease modifying effect was associated with a reduced MFS, which is a pathological hallmark of TLE and commonly observed in this model (Schulz et al., 2012). However, MFS in TBB-pretreated epileptic rats did not differ from MFS in vehicle-pretreated epileptic animals (Figure 2).

### TBB Facilitates Short-Term Synaptic Plasticity in Epileptic Animals

**FIGURE 3:**
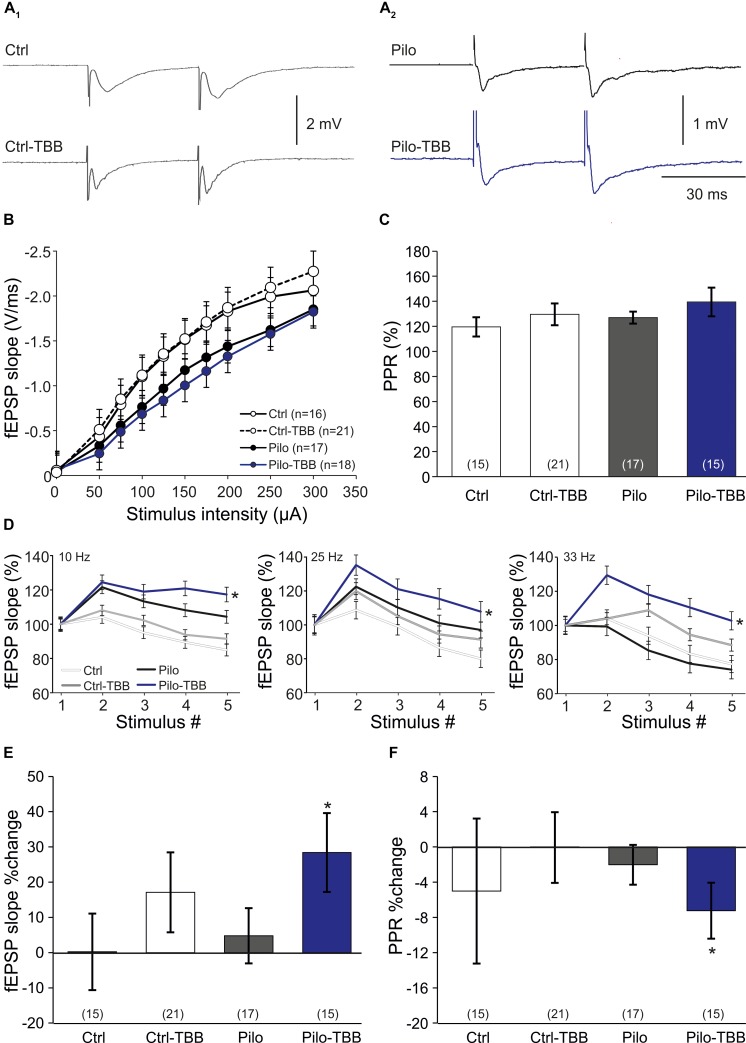
CK2 inhibition facilitates CA1 plasticity. **(A)** Sample traces of CA1 stratum radiatum field excitatory postsynaptic potentials (fEPSP) following Schaffer collateral stimulation in control (A_1_) and epileptic animals (A_2_). **(B)** Input–output curves of all four groups without significant group differences. **(C)** Paired-pulse ratios (PPRs) were similar among all four experimental groups. Short-term plasticity during five stimuli at frequencies of 10, 25, and 33 Hz **(D)**, and subsequent changes of fEPSP slope **(E)** and PPR **(F)** after this short-term plasticity paradigm. Only slices from the TBB-pretreated epileptic group showed significantly facilitated plasticity. *P < 0.05.

Our data so far indicate that *in vivo* TBB pretreatment affects pilocarpine-induced SE and exerts disease modifying properties during epileptogenesis. Since TBB was not continued after SE, we hypothesized that TBB leads to persistent changes in the epileptic, but probably also in the control tissue. In order to address this issue, we first analyzed extracellular field potentials in control and post-SE tissue, both taken from either vehicle- or TBB-pretreated animals. Basic properties of synaptic transmission such as input–output curves or paired-pulse plasticity were not altered by TBB (Figures 3A–C). In contrast, short-term plasticity protocols of five stimuli at increasing frequencies showed significantly higher potentiation in TBB-pretreated epileptic animals as compared to both vehicle-pretreated epileptic tissue as well as tissue from control rats (both comparisons: *P* < 0.05, three-way ANOVA followed by Holm–Sidak test; Figure 3D; no significant interaction between stimulus number and animal group or between stimulus number and pretreatment). After delivering the short-term plasticity protocols, the field potentials were significantly increased in tissue from TBB-pretreated epileptic animals (*P* < 0.05 versus baseline, Wilcoxon signed rank test; Figure 3E) indicating that some degree of long-term plasticity might have been elicited by these short protocols. Concomitantly with this potentiation, the PPR was significantly reduced (*P* < 0.05, Wilcoxon signed rank test; Figure 3F) suggesting presynaptic mechanism for this plasticity-facilitating effect of TBB in tissue from chronically epileptic rats. These results demonstrate that TBB may lead to persistent changes in the synaptic network of the CA1 area which may be even more pronounced in epileptic than in control tissue.

### TBB Reduces the Number of Burst Firing Neurons in CA1

**FIGURE 4:**
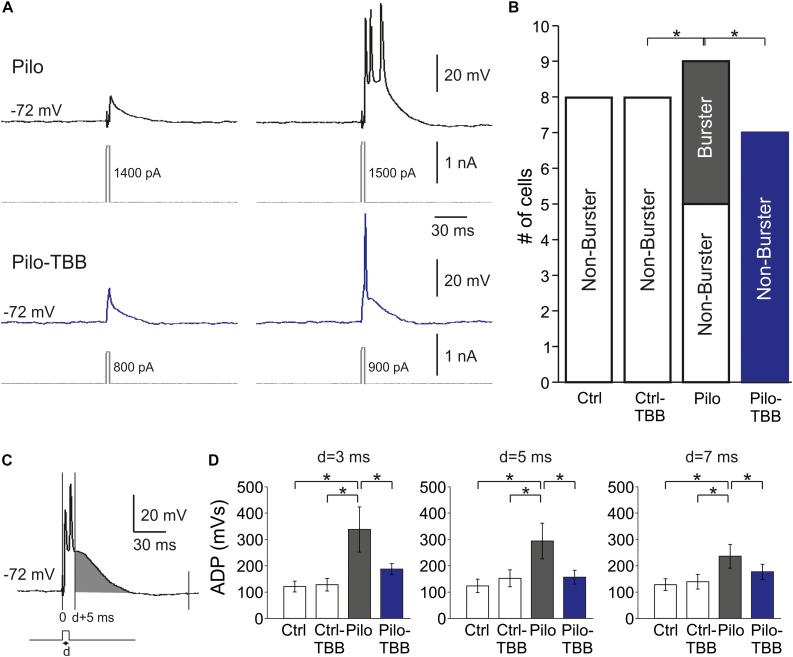
CK2 inhibition reduces bursting in CA1. **(A)** Sample traces of CA1 pyramidal cell intracellular recordings from a vehicle-pretreated epileptic rat (Pilo, black traces) and a TBB-pretreated epileptic rat (Pilo-TBB, blue traces). Note the bursting of the upper traces upon just suprathreshold stimulation. **(B)** Proportions of burster and non-burster neurons in the four experimental groups (*P < 0.05). **(C,D)** Afterdepolarizing potential (ADP) with different pulse durations (*d* = 3, 5, 7 ms). The area under curve of the ADP (starting from *d* + 5 ms, indicated in gray in **C**) of cells from vehicle-pretreated epileptic animals (Pilo) was significant against the ADP of all other experimental groups (*P < 0.05, three-way ANOVA with Tukey *post hoc* test).

One hallmark of chronically epileptic tissue is the presence of burst firing neurons, especially in CA1 (Chen et al., 2011). To study this issue in more detail, we performed intracellular recordings from CA1 neurons in vehicle- or TBB-pretreated control and epileptic animals. A typical burst firing behavior is depicted in Figure 4A, obtained from a neuron of a vehicle-pretreated animal (upper trace in gray), showing three action potentials for the lowest suprathreshold depolarizing current injection. In contrast, a regular firing behavior is shown in the lower trace in Figure 4A (blue trace), taken from a TBB-pretreated animal. Burst firing was observed in 4/9 neurons from vehicle-pretreated epileptic animals, but in 0/7 neurons from TBB-pretreated epileptic animals (*P* < 0.05, χ^2^ test; Figure 4B). In control rats (either vehicle- or TBB-pretreated), no burst firing neurons were observed (Figure 4B).

Bursting behavior may be the consequence of a prolonged afterdepolarizing potential (ADP; Chen et al., 2011). We therefore determined the AUC of the ADP starting at 5 ms after the depolarizing step (Figure 4C). The duration of the depolarizing step (“d” in Figures 4C,D) was set to 3, 5, and 7 ms, and the ADP was significantly larger in the vehicle-pretreated epileptic group as compared to both TBB-pretreated epileptic and control groups (*P* < 0.05, three-way ANOVA; Figure 4D). Taken together, pilocarpine-induced SE caused a significant increase in ADP size probably involved in burst firing behavior which in turn was restored by TBB pretreatment *in vivo* prior to SE.

### TBB Modifies K_Ca_2 Channel Function

**FIGURE 5:**
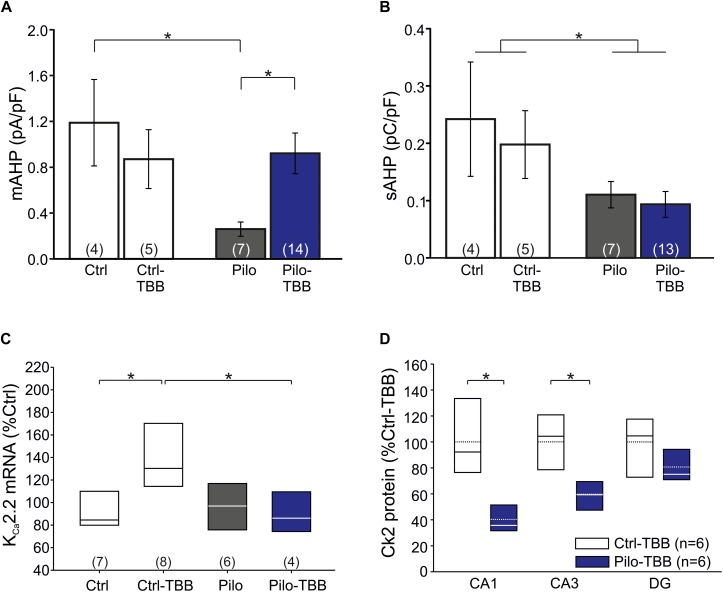
CK2 inhibition modifies KC2 function. **(A)** Bar graph of the mAHP-mediating current in all four experimental groups. CA1 cells from vehicle-pretreated epileptic animals (Pilo) showed significantly less mAHP currents than control cells and cells from TBB-pretreated epileptic animals. **(B)** The sAHP charge transfer is significantly smaller in epileptic animals than in controls. TBB has no effect on the sAHP charge transfer. **(C)** Quantitative RT-PCR analysis of the CA1 subfield reveals that tissue from TBB-pretreated control animals contained significantly more K_Ca_2.2 mRNA than vehicle-pretreated controls and TBB-pretreated epileptic rats. **(D)** Western blot analysis of CK2 protein in TBB-pretreated tissue. Note the region-specific differences between control and epileptic tissues. *P < 0.05.

The significant reduction of the number of burst firing neurons in the epileptic CA1 after TBB pretreatment following ADP normalization is an attractive molecular mechanism for the observed disease modifying properties *in vivo*. Since we previously demonstrated that K_Ca_2.2 was downregulated in isolated CA1 neurons from chronically epileptic tissue and TBB administered during chronic epileptic stages enhanced K_Ca_2.2 levels in CA1 from post-SE animals (Schulz et al., 2012; Bajorat et al., 2018), we first performed patch-clamp recordings from visualized CA1 pyramidal neurons in hippocampal slices and confirmed the impaired function of K_Ca_2 by recording the mAHP-mediating current density (0.26 ± 0.06 pA/pF,*n* = 7 versus 1.19 ± 0.38 pA/pF,*n* = 4, *P* < 0.05, unpaired *t*-test, Figure 5A) as previously reported in isolated cells (Schulz et al., 2012). Pretreatment with TBB prior to SE significantly enhanced the mAHP current density (0.92 ± 0.18 pA/pF,*n* = 14, *P* < 0.05, unpaired *t*-test; Figure 5A) suggesting that TBB might indeed enhance K_Ca_2 function. We next analyzed the sAHP, for which the underlying ion channels are less clearly determined, but do not seem to involve K_Ca_2 (Bond et al., 2004; Villalobos et al., 2004; Pedarzani et al., 2005). Consistent with this, the sAHP charge transfer as assessed by determining the AUC of the sAHP-mediating current density was significantly reduced in CA1 neurons from chronically epileptic rats as compared to control tissue (*P* < 0.05, two-way ANOVA), but without any significant TBB effect (Figure 5B).

From these findings so far, it is conceivable that K_Ca_2 function is reduced in chronically epileptic CA1 neurons, and we speculated that TBB might upregulate K_Ca_2.2 gene expression (Bajorat et al., 2018). We, therefore, performed real-time RT-PCR and, as expected, K_Ca_2.2 mRNA was significantly upregulated in TBB-pretreated control animals (*P* < 0.05, ANOVA and Duncan *post hoc* test; Figure 5C). More importantly, in TBB-pretreated animals, SE was associated with a significant reduction of K_Ca_2.2 expression as previously observed (Schulz et al., 2012; *P* < 0.05, ANOVA and Duncan *post hoc* test; Figure 5C). However, mRNA levels of K_Ca_2.2 were not different between Pilo and Pilo-TBB groups. Hence, the TBB effect on the mAHP in epileptic tissue cannot be explained by simple upregulation of K_Ca_2.2 in CA1 neurons. Based on the idea that K_Ca_2.2 function is reduced by CK2 activity (Bildl et al., 2004; Allen et al., 2007), we performed Western blotting of CK2 protein in six control and six epileptic TBB-pretreated animals (Figure 5D). These analyses indeed showed a region-specific lack of CK2 protein in epileptic animals which was most pronounced in CA1 (40 ± 5% of Ctrl-TBB tissue, *P* < 0.002, *t*-test; Figure 5D) suggesting a persistent downregulation of this enzyme in this area as a potential mechanism for the disease modifying effect.

### TBB Modifies HCN Channel Function

**FIGURE 6:**
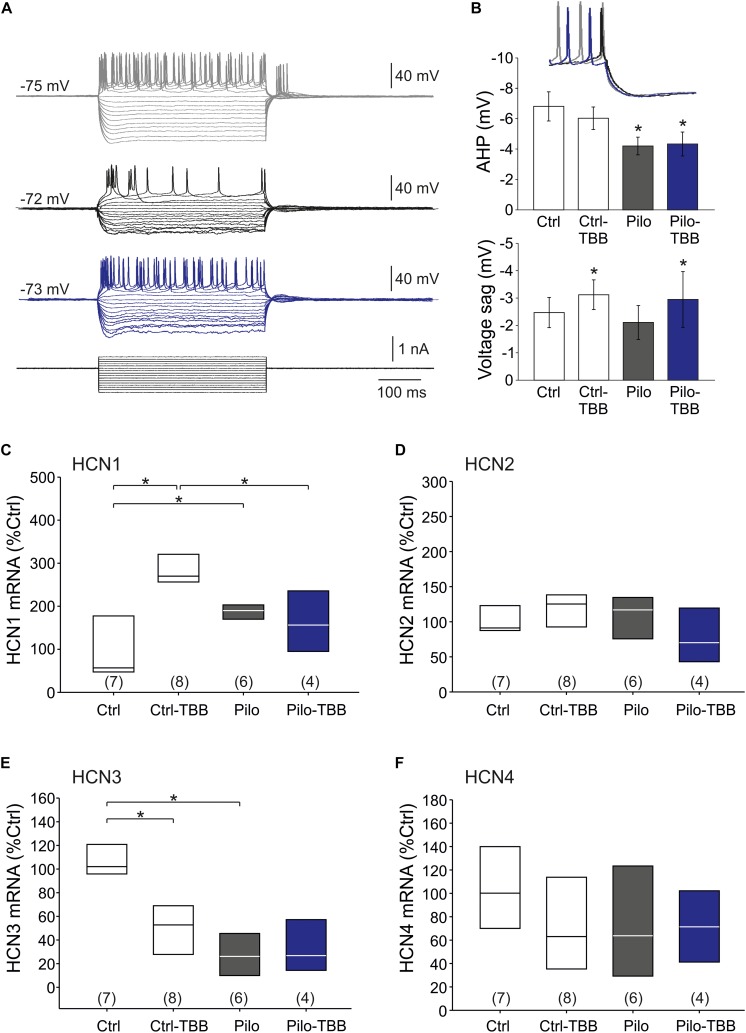
CK2 inhibition modifies HCN channel function. **(A)** Sample traces of the membrane potential following hyperpolarizing and depolarizing pulse steps in intracellular recordings from CA1 neurons (gray: control, black: vehicle-pretreated epileptic, blue: TBB-pretreated epileptic). **(B)** Bar graphs of the train-AHP amplitude and voltage sag obtained from recordings shown in **A**. The train-AHP was significantly smaller in epileptic tissues regardless of the treatment with TBB. In contrast, the voltage sag was enhanced in TBB-pretreated tissue from both control and epileptic animals. Quantitative RT-PCR analysis of the CA1 subfield of all four HCN channel isoforms revealed significant and inverse expression changes for HCN1 **(C)** and HCN3 **(E)**, but no changes for HCN2 **(D)** and HCN4 **(F)**. *P < 0.05.

The findings so far point to an impaired function of K_Ca_2 in the epileptic CA1 area. Nonetheless, some data appeared inconclusive. Therefore, we asked whether the AHP which is a prominent K_Ca_2.2 function (Hotson and Prince, 1980; Bond et al., 2004) might be altered following SE and TBB pretreatment. To this end, we performed intracellular recordings from CA1 pyramidal cells in vehicle- and TBB-pretreated epileptic and control animals (Figure 6A). Basic passive membrane properties such as RMP, membrane resistance and time constant were not different in these four groups (Table 2). With respect to the AHP, we found that the AHP following a train of action potentials (train-AHP) was significantly reduced in epileptic CA1 neurons (−4.2 ± 0.6 mV,*n* = 10 versus −6.8 ± 1.0 mV,*n* = 9, *P* < 0.05, two-way ANOVA with Tukey *post hoc* test; Figures 6A,B) consistent with a previous report (Schulz et al., 2012). However, TBB pretreatment had no significant effect on these values (Figure 6B). Instead, we observed a prominent voltage sag in the individual voltage traces in cells from controls (gray traces in Figure 6A) as well as TBB-pretreated epileptic rats (blue traces in Figure 6A), but not from vehicle-pretreated epileptic rats (black traces in Figure 6A) suggesting that TBB might modify HCN channel function. Indeed, there was a significant enhancement of the voltage sag following TBB pretreatment in both control (3.0 ± 1.4 mV,*n* = 10 versus 2.1 ± 1.0 mV,*n* = 10, *P* < 0.05, two-way ANOVA with Tukey *post hoc* test; Figure 6B) and epileptic animals (3.1 ± 2.9 mV,*n* = 9 versus 2.0 ± 2.1 mV,*n* = 10, two-way ANOVA with Tukey *post hoc* test; Figure 6B). Thus, we repeated the gene expression analysis with all HCN isoforms (HCN1-4; Figures 6C–F). In control animals, TBB led to differential alteration of HCN transcription with a significant upregulation of HCN1 (288 ± 27%,*n* = 8, *P* < 0.05, ANOVA with Duncan *post hoc* test; Figure 6C) and concomitantly, a significant downregulation of HCN3 (51 ± 10%,*n* = 8, *P* < 0.05, ANOVA with Duncan *post hoc* test; Figure 6E). Interestingly, epileptic animals showed similar differential alterations when compared to controls (HCN1 up-regulated to 187 ± 9% and HCN3 downregulated to 28 ± 8%,*n* = 6, *P* < 0.05, ANOVA with Duncan *post hoc* test; Figures 6C,E). In epileptic animals, however, TBB pretreatment before SE did not significantly alter HCN1 or HCN3 expression levels (Figures 6C,E), and for the other two isoforms, HCN2 and HCN4, no significant changes were observed at all (Figures 6D,F). Taken together, this RT-PCR analysis indicates that TBB leads to differential expression changes of HCN channels which might have contaminated our analyses on K_Ca_2 channel function.

**TABLE 2:** Intrinsic cellular parameters.

	**Vehicle-pretreated control rats (Ctrl)**	**TBB-pretreated control rats (Ctrl-TBB)**	**Vehicle-pretreated epileptic rats (Pilo)**	**TBB-pretreated epileptic rats (Pilo-TBB)**
RMP (mV)	−58.9 ± 1.2(*n* =12)	−60.0 ± 1.2(*n* =12)	−59.3 ± 2.1(*n* =9)	−57.3 ± 2.2(*n* =12)
	−69.6 ± 5.5 (*n* = 5)*	−72.1 ± 2.1 (*n* = 5)**	−73.9 ± 4.1 (*n* = 6)**	−68.1 ± 5.5 (*n* = 5)
R_*m*_ (MΩ)	77.4 ± 9.7(*n* =11)	65.3 ± 7.1(*n* =10)	58.8 ± 8.0(*n* =10)	63.5 ± 5.5(*n* =9)
	81.6 ± 10.6 (*n* = 5)	95.7 ± 8.8 (*n* = 5)**	98.5 ± 7.2 (*n* = 6)*	88.3 ± 11.0 (*n* = 6)
τ _*m*_ (ms)	8.5 ± 1.0(*n* =11)	8.9 ± 0.7(*n* =10)	8.9 ± 0.7(*n* =10)	8.2 ± 0.6(*n* =9)
	11.0 ± 2.0 (*n* = 5)	25.1 ± 2.6 (*n* = 5)**	23.2 ± 3.6 (*n* = 6)**	23.5 ± 3.0 (*n* = 6)**

Values obtained in the presence of ZD7288 are given in gray. *P*-values (*P < 0.05; **P < 0.01) correspond to unpaired *t*-test or Mann–Whitney *U*-test, respectively.

### TBB Enhances Train-AHP and Reduces Excitability in HCN-Blocking Conditions

**FIGURE 7:**
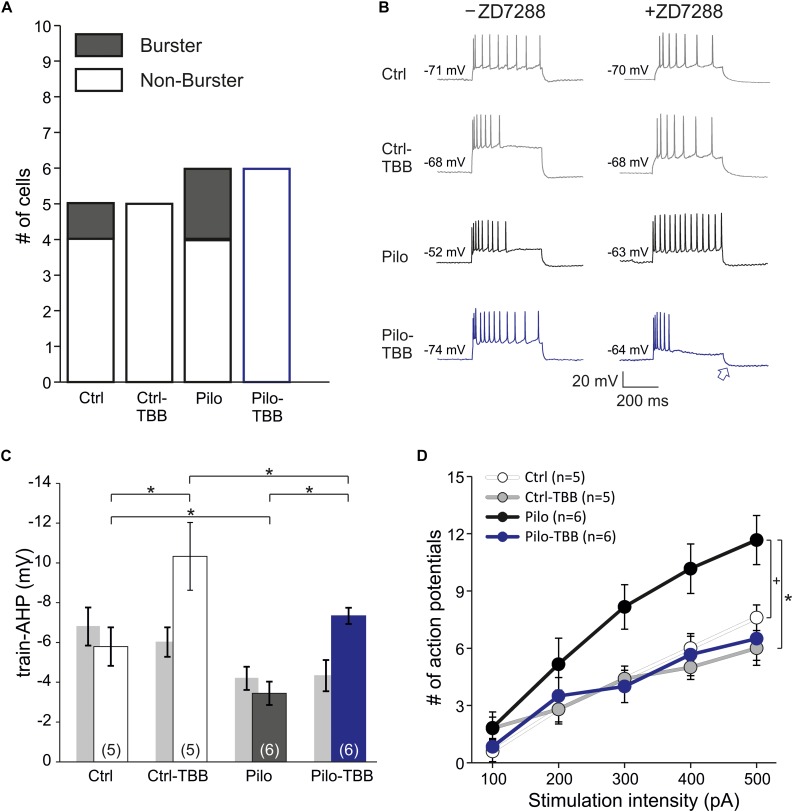
CK2 inhibition enhances train-AHP and reduces excitability in HCN-blocking conditions. **(A)** Proportions of burster and non-burster neurons in the four experimental groups in the presence of the HCN channel blocker ZD7288. **(B)** Sample traces of intracellular recordings following prolonged depolarization (600 ms) used for train-AHP amplitude (e.g., see blue arrow) and number of action potentials. For comparison, sample traces in the absence of ZD7288 were also given (left side). **(C)** Bar graph of the train-AHP amplitude shows a significantly increased train-AHP in cells from TBB-pretreated epileptic rats (blue bar graph). For the sake of clarity, the train-AHP in the absence of ZD7288 (gray bar graphs, values from Figure 5B) are also shown. Significant effects were detected between animal groups (Ctrl versus Pilo, *P < 0.05) and pretreatment groups (vehicle versus TBB, *P < 0.05), but not for the ZD7288 effect by three-way ANOVA with Holm–Sidak *post hoc* tests. However, there was a significant interaction between ZD7288 and pretreatment with TBB or vehicle (*P < 0.05). **(D)** Number of action potentials during 600 ms depolarization (from 100 to 500 pA). Two-way ANOVA with Tukey *post hoc* tests showed a trend effect between animal groups (Ctrl versus Pilo, ^+^*P* = 0.08) and a significant effect treatment groups (vehicle versus TBB, *P < 0.05).

In order to rule out contamination of differential HCN expression changes following TBB pretreatment, we repeated intracellular recordings in the presence of the HCN channel blocker ZD7288 (20 μM, Figure 7). Under these conditions, the proportion of burst firing neurons in the epileptic CA1 area was reduced, but still present (two of six cells) and no longer significant when compared to CA1 neurons from TBB-pretreated epileptic rats (zero of six cells, Figure 7A) suggesting that both HCN channel and K_Ca_2 function contribute to bursting behavior. However, the power of this analysis is insufficient to dissect a differential TBB effect on those channels with respect to bursting, in particular with respect to other candidate mechanisms such as persistent sodium currents (Chen et al., 2011). Intriguingly, in contrast to the train-AHP analysis above in the absence of ZD7288, prolonged depolarization led to a markedly increased train-AHP in TBB-pretreated epileptic animals under HCN-blocking conditions (see blue traces and blue arrow in Figure 7B). On average, the train-AHP in this group was −5.7 ± 1.5 mV (*n* = 6, blue bar in Figure 7C) and thus matched with control levels (−5.8 ± 1.0 mV,*n* = 5). The statistical comparison by three-way ANOVA revealed a significant animal effect (Ctrl versus Pilo, *P* < 0.05, ANOVA with Holm–Sidak *post hoc* test) and significant pretreatment effect (vehicle versus TBB, *P* < 0.05, ANOVA with Holm–Sidak *post hoc* test), but no significant effect of ZD7288 (Figure 7C). However, there was a significant interaction between ZD7288 and pretreatment with TBB or vehicle (*P* < 0.05, three-way ANOVA) indicating that the ZD7288 effect depends on the type of pretreatment. The sample traces in Figure 7B also suggested an impaired spike frequency adaptation in CA1 neurons from vehicle-treated epileptic animals when studied in the presence of ZD7288 (black traces in Figure 7B). As shown in Figure 7D, the mean number of action potentials during the 400 ms pulse was substantially higher in this group, and two-way ANOVA showed a trend effect by animal (Ctrl versus Pilo, *P* = 0.08, indicated by “+,” ANOVA with Tukey *post hoc* test) and a significant effect by treatment (vehicle versus TBB, *P* < 0.05, ANOVA with Tukey *post hoc* test). Hence, TBB appears to modify both K_Ca_2 and HCN channel function with partly opposing effects. We conclude that under HCN-blocking conditions, TBB treatment prior to SE leads to persistent increase of the train-AHP amplitude and spike frequency adaptation in epileptic animals, both of which are prominent K_Ca_2 functions.

## Discussion

### Disease Modification After Transient CK2 Inhibition *in vivo*

In the present study, we investigated the effects of a short-term *in vivo* CK2 inhibition with TBB prior to pilocarpine-induced SE. First, we found that the first generalized seizure after pilocarpine injection was delayed, and moreover, the epileptic phenotype as assessed by EEG spike and video seizure rate analyses was altered in TBB-pretreated epileptic animals. Interestingly, the typical seizure cluster pattern observed in almost half of the chronically epileptic rats in this model (Bajorat et al., 2011, 2016, 2018) was less frequently detected in the TBB-pretreated group, and TBB slowed down disease progression during epileptogenesis. Since TBB was not effective as an anti-seizure drug when administered after epileptogenesis (Bajorat et al., 2018), these data indicate that TBB has potential disease modifying properties. In addition, Western blot analysis demonstrated that CK2 protein abundance was significantly reduced in TBB-pretreated epileptic tissue, especially in CA1. Interestingly, water maze training significantly reduced CK2 activity in the CA1 area, but not in the dentate gyrus (Chao et al., 2007) indicating region-specific regulation of this enzyme. This might be relevant since CA1 long-term potentiation was found to be increased after water maze training (Rehberg et al., 2017).

If CK2 is less abundant, what might be the consequences in the epileptic CA1 neuron? CaM is phosphorylated by CK2 and, in turn, dephosphorylated by protein phosphatase 2A (Bildl et al., 2004; Allen et al., 2007). The phosphomimetic T80D-CaM species decreased Ca^2+^ sensitivity and reduced K_Ca_2 function (Allen et al., 2007). Hence, decreased CK2 activity and concomitantly predominant activity of protein phosphatase 2A would increase K_Ca_2 function without necessarily altering transcription. In the present study, we observed that TBB increased K_Ca_2.2 transcription in control (Bajorat et al., 2018), but not in epileptic animals. This is an intriguing finding probably indicating that epileptogenesis could have reversed the K_Ca_2.2 upregulation. Although we have not performed a serial analysis of K_Ca_2.2 transcription directly after SE as well as during epileptogenesis, we assume that ongoing epileptic activity might decrease K_Ca_2 function since chronically epileptic rats have significantly less K_Ca_2.2 protein (Schulz et al., 2012). However, we did not detect a significant transcriptional downregulation of K_Ca_2.2 in vehicle-pretreated epileptic rats as found in our previous paper (Schulz et al., 2012), because we used three different housekeeping genes and averaged the results. Hence, the TBB-induced upregulation of K_Ca_2.2 in control tissue was indeed confirmed (Bajorat et al., 2018), but reduced K_Ca_2.2 protein levels in chronically epileptic tissue (Schulz et al., 2012) are probably due to internalization and degradation observed at potentiated synapses (Lin et al., 2008) as well as after epileptiform activity (Kernig et al., 2012; Müller et al., 2018) rather than transcriptional downregulation. Furthermore, it is important to note that the K_Ca_2.3 isoform which may also be relevant in CA1 and dentate gyrus (Ballesteros-Merino et al., 2014) was not found to be reduced in the chronically epileptic CA1 (Schulz et al., 2012) suggesting that the presumed reduction of CK2 activity should enhance K_Ca_2.3 function as well. Taken together, our findings are consistent with a persistently diminished CK2 activity leading to enhanced K_Ca_2 function in CA1 as a potentially relevant mechanism for the disease modifying effect of TBB.

Are there alternative molecular pathways for the disease modifying effect of TBB? On the one hand, we found an increased number of burst-spiking neurons in CA1 of vehicle-pretreated, but not in CA1 of TBB-pretreated epileptic rats. The ADP and burst-firing behavior have been recognized as common intrinsic properties of epileptic tissue, especially in the subiculum and CA1 (Magee and Carruth, 1999; Su et al., 2001; Yue and Yaari, 2004; van Welie et al., 2006; Jarsky et al., 2008). Based on this literature, the ADP was demonstrated to be due to persistent Na^+^ and Ni^+^-sensitive Ca^2+^ currents, rather than Ca^2+^-activated K^+^ currents (Magee and Carruth, 1999; Su et al., 2001). However, pharmacological inhibition of KCNQ/M-type K^+^ currents increased spike ADP and burst-spiking in CA1 (Yue and Yaari, 2004) suggesting that K^+^ currents can substantially modify these intrinsic firing properties. Thus, the effect of TBB pretreatment to prevent burst-firing behavior might indicate that Ca^2+^-activated K^+^ channels and probably further ion channels have been modified by TBB pretreatment. In this respect, it is noteworthy that functional HCN3 upregulation by phosphatidylinositol-4,5-bisphosphate facilitated low-threshold burst-firing in thalamic neurons (Ying et al., 2011). Hence, one might speculate whether the HCN3 downregulation found in TBB-pretreated epileptic tissue could have exerted the opposite effect and inhibited the development of burst-firing. On the other hand, we observed at least one control burster cell in the presence of ZD7288, and increased excitability has been associated with downregulation of HCN channels in chronically epileptic CA1 (Adams et al., 2009). Nonetheless, there is also uncertainty about the selectivity of TBB which will inhibit other kinases, too (Pagano et al., 2008). CK2 has a pretty large number of substrates (Meggio and Pinna, 2003), and recently, CK2 was demonstrated to phosphorylate histone chaperone Spt6 that is associated with RNA polymerase II and therefore promotes DNA transcription (Dronamraju et al., 2018). Since CK2 is an oncogenic enzyme involved in epigenetic processes (reviewed by Gowda et al., 2019), further targets need to be unraveled. Here, we focused on K_Ca_2 channel-mediated mechanisms.

### Increased K_C__a_2 Function as the Relevant Disease Modifying Mechanism

On the network level, we did not observe significant changes of basal synaptic transmission after TBB pretreatment indicating that a potential K_Ca_2-mediated reduction of the EPSP (Hammond et al., 2006) did not occur in our hands because postsynaptic Ca^2+^ increase during basal transmission was quite low. Instead, short-term plasticity was significantly facilitated and PPRs were changed in potentiated synapses in TBB-pretreated epileptic animals. These findings might point to a presynaptic mechanism, but K_Ca_2.2 channels were excluded to have relevant presynaptic functions (Hammond et al., 2006).

Hence, what is the evidence for an enhanced K_Ca_2 function in TBB-pretreated epileptic CA1? Intrinsic cellular properties such as the mAHP and the spike frequency adaptation are well-established K_Ca_2 functions of CA1 neurons (Bond et al., 2004; Pedarzani et al., 2005; Hammond et al., 2006; Schulz et al., 2012). First, we could demonstrate that mAHP-mediating K^+^ currents were significantly enhanced by TBB pretreatment in tissue from epileptic animals. On the other hand, the sAHP was also reduced in epileptic CA1 neurons, but not altered by TBB treatment. This is consistent with the view that K_Ca_2 is not involved in sAHP (Stocker et al., 1999; Bond et al., 2004; Villalobos et al., 2004; Pedarzani et al., 2005), but the molecular sAHP-mediating candidates are less clear and may certainly involve HCN channels (Gu et al., 2005; Kaczorowski, 2011). Importantly, we observed that HCN channel function was enhanced in TBB-pretreated epileptic tissue. In addition, the transcriptional analysis revealed HCN1 upregulation and HCN3 downregulation in epileptic CA1. This is in contrast to previous reports showing HCN1 downregulation in the pilocarpine model (Jung et al., 2007, 2010), but more recently, HCN1 immunoreactivity was found be increased, especially in CA1 interneurons (Oh et al., 2012). Moreover, HCN1 upregulation was also found in the epileptic dentate gyrus (Bender et al., 2003; Surges et al., 2012). Hence, HCN isoform expression and protein abundance may depend on the cell type. Nonetheless, since pharmacological HCN channel inhibition by ZD7288 that does not distinguish between HCN isoforms led to an increase of sAHP-mediating currents (Gu et al., 2005), we propose that a net enhancement of HCN channel function by TBB could have antagonized an expected TBB-mediated sAHP increase due to enhanced K_Ca_2 function. In fact, we previously found that the sAHP was increased in control and epileptic tissue when animals were pretreated *in vivo* with TBB (Brehme et al., 2014). On the one hand, this could be simply due to different recording procedures (isolated cells versus slice experiments) that differ with respect to recording from dendritic compartments expressing high levels of HCN1 (Nolan et al., 2004). On the other hand, however, this discrepancy may indicate that enhanced HCN channel function following CK2 inhibition may occur with delayed time course and, probably, activity-dependent (Shin and Chetkovich, 2007). This is an attractive mechanism to be addressed in further studies.

Second, we addressed K_Ca_2 function under HCN channel-blocking conditions. In the presence of the non-selective inhibitor ZD7288, the train-AHP recorded with intracellular electrodes was significantly reduced in vehicle-treated epileptic tissue, but could be enhanced by TBB pretreatment in both control and epileptic animals. This is an important finding, because HCN inhibition shifts the RMP to hyperpolarizing values which should rather decrease the driving force for K_Ca_2 channels. On the other hand, membrane resistance also depends on HCN channel function (Surges et al., 2004), and ZD7288 increased the resistance—and thus could have counteracted the probably reduced K^+^ currents. Hence, the enhanced train-AHP recorded intracellularly confirmed the membrane current recordings obtained with the patch-clamp method.

Third, K_Ca_2 channels also play a major role in spike frequency adaptation (Pedarzani et al., 2005). In the present study, we studied spike frequency adaptation in HCN channel-blocking conditions and could demonstrate that this phenomenon was impaired in chronically epileptic tissue confirming previous results in isolated neurons (Schulz et al., 2012). Moreover, TBB restored spike frequency adaptation in epileptic tissue which can be viewed as an important antiepileptic phenomenon. In summary, our data strongly suggest that CA1 neurons from TBB-pretreated epileptic animals show a significantly enhanced K_Ca_2 function that likely contributes to disease modification.

## Conclusion

Our experiments demonstrated that TBB pretreatment prior to SE led to reduced epileptogenesis and to differential gene expression changes of K_Ca_2.2, but also of HCN1 and HCN3 channels. Under HCN channel blocking conditions, pretreatment with TBB could significantly enhance K_Ca_2 channel functions such as the AHP and the spike frequency adaptation in epileptic animals, probably due to a persistently decreased CK2 protein abundance.

## Data Availability Statement

The datasets generated for this study are available on request to the corresponding author.

## Ethics Statement

The animal study was reviewed and approved by Landesamt für Landwirtschaft, Lebensmittelsicherheit und Fischerei Mecklenburg-Vorpommern (numbers M-V/TSD/7221.3-1.1-013/10 and 7221.3-1.1-019/13).

## Author Contributions

FS, SM, XG, LS, HB, and TR performed the experiments. FS, TK, and RK contributed to conception and design of the study. FS, SR, AE, and RK organized the database. FS, SM, LS, MR, DG, TK, and RK performed the statistical analysis. TK wrote the first draft of the manuscript. FS, SM, MR, and RK wrote the sections of the manuscript. LS, MR, and DG contributed to manuscript preparation. All authors contributed to manuscript revision and read and approved the final version of this manuscript for submission.

## Conflict of Interest

The authors declare that the research was conducted in the absence of any commercial or financial relationships that could be construed as a potential conflict of interest.
